# The Address in Surgery

**Published:** 1901-08-03

**Authors:** 


### THE ADDRESS IN SURGERY.

Sir William Thomson, C.B., M.D., F.R.C.S.I., delivered the Address in Surgery, taking for his subject "Some Surgical Lessons from the Campaign in South Africa." He dealt first with the general effect of modern small-bore fire and then went on to consider the wounds produced by the small-bore bullet in the soft parts, in the bones, in the joints, and in the abdomen. In regard to the examination of wounds, he said that the treatment in the first stage of wounds involving bones had led to some difference of opinion among surgeons, but he is satisfied that reason and experience point in one direction only, namely, that local examination ought not to be carried out unless the surgeon should be driven to it by developments. "With rare exceptions the wrong thing to do on the battle-field, in a recent wound involving fracture of a bone is to explore it with probes, or to enlarge it for the passage of a finger and the removal of fragments." The war is still in progress, he said, and will take a considerable time after its conclusion before we can make an effective summing up. But even thus far we have materials for conclusions which are not likely to be much modified. We have seen a marvellous proportion of recoveries from severe wounds, and we have been able to rejoice at the great reduction of suppurating cases, and the practical absence of such infective diseases as tetanus, erysipelas, pyæmia and hospital gangrene. This great difference in the records of to-day as compared with those of older and more bloody wars does not depend mainly upon the alteration in the bullet used. That, as we have seen, can do fearful things to a dense obstacle, and add to its power for mischief by the laceration caused by bony fragments which it scatters on impact. To some extent, the smallness of the canal made by it helps towards recovery. But, above all, the results are due to the treatment of the wounds by the surgeon, and the operation of those great principles which have developed under the splendid genius of Lord Lister. When the recoveries from wounds in this war are made up and analysed, it will be seen what a tremendous progress has been made in protecting the injured from disaster, even in the face of all the difficulties of the battlefield. For, while in the general and stationary hospitals the precautions necessary in modern surgery can be observed, it is, after all, at the initial stage that safety from sepsis is to be secured. "The fate of the wounded rests in the hands of the one who applies the first dressing," wrote Von Nussbaum, and the truth of this assertion has been proved every day.

War is at all times full of horrors, but as it is waged to-day it is less terrible than before, a result in part no doubt due to the smaller bullet employed, but more certainly to the improved methods which have been established in the practice of surgery.

Our profession has taken its full share in the work in South Africa, and whether as officers of the Royal Army Medical Corps or as civil surgeons, all have worked ceaselessly in ministering to the sick and wounded. They have not flinched in danger on the field, and they have vied with their combatant fellows in deeds of valour.

